# In vitro susceptibility of four antimicrobials against *Riemerella anatipestifer* isolates: a comparison of minimum inhibitory concentrations and mutant prevention concentrations for ceftiofur, cefquinome, florfenicol, and tilmicosin

**DOI:** 10.1186/s12917-016-0796-3

**Published:** 2016-11-09

**Authors:** Yafei Li, Yanan Zhang, Huanzhong Ding, Xian Mei, Wei Liu, Jiaxiong Zeng, Zhenling Zeng

**Affiliations:** Guangdong Provincial Key Laboratory of Veterinary Pharmaceutics Development and Safety Evaluation, South China Agricultural University, Guangzhou, 510642 People’s Republic of China

**Keywords:** *R. anatipestifer*, Minimum inhibitory concentration, Mutant prevention concentration

## Abstract

**Background:**

Mutant prevention concentration (MPC) is an alternative pharmacodynamic parameter that has been used to measure antimicrobial activity and represents the propensities of antimicrobial agents to select resistant mutants. The concentration range between minimum inhibitory concentration (MIC) and MPC is defined as mutant selection window (MSW). The MPC and MSW parameters represent the ability of antimicrobial agents to inhibit the bacterial mutants selected. This study was conducted to determine the MIC and MPC values of four antimicrobials including ceftiofur, cefquinome, florfenicol and tilmicosin against 105 *Riemerella anatipestifer* isolates.

**Results:**

The MIC_50_/MIC_90_ values of clinical isolates tested in our study for ceftiofur, cefquinome, florfenicol and tilmicosin were 0.063/0.5、0.031/0.5、1/4、1/4 μg/mL, respectively; MPC_50_/ MPC_90_ values were 4/64、8/64、4/32、16/256 μg/mL, respectively. These results provided information on the use of these compounds in treating the *R. anatipestifer* infection; however, additional studies are needed to demonstrate their therapeutic efficacy.

**Conclusion:**

Based on the MSW theory, the hierarchy of these tested antimicrobial agents with respect to selecting resistant subpopulations was as follows: cefquinome > ceftiofur > tilmicosin > florfenicol. Cefquinome was the drug that presented the highest risk of selecting resistant mutant among the four antimicrobial agents.

## Background

*Riemerella anatipestifer* has been one of the most troublesome etiological agents and causes heavy loss in duck industry. It occurs worldwide, especially in Southeast Asia [1]. The existence of *R. anatipestifer* infection symptoms observed is characterized by fibrinous pericarditis, perihepatitis, airsacculitis, caseous salpingitis, and meningitis. To date, at least twenty-one serotypes of *R. anatipestifer* have been identified and little cross-immunoprotection among serotypes was reported [2]. People have been making great efforts to find new strategies to prevent or control *R. anatipestifer* infection, since much work has been done concentrating on the identification of factors associated with virulence [1, 3, 4] and immunogenic characterization based on outer membrane protein A in recent years [5–7]. Even so, chemotherapy is still a major approach in the treatment of *R. anatipestifer* infection because of the complex immunology situation currently. Due to the concern of high incidence of *Riemerella anatipestifersis* and increasingly severe drug resistance or reduction of susceptibility, obtaining new treatment information and promising results with antimicrobial agents seems necessary [8–10].

Ceftiofur (β-lactam), cefquinome (β-lactam), florfenicol (phenicol), and tilmicosin (macrolide) belong to three families of antimicrobial agents and were developed for exclusive use in animals. They have exhibited remarkable antibacterial effects against diverse microorganisms since being introduced, although resistance to those drugs mentioned above has also been reported [11–14]. The escalating resistance of *R. anatipestifer* field strains and concerns over animals as putative reservoirs for antimicrobial resistance genes force us to develop strategies to make full use of the current drugs [8, 15].

Traditionally, the in vitro activity of antimicrobial susceptibility is assessed by minimum inhibitory concentration (MIC) or minimum bactericidal concentration (MBC). The mutant prevention concentration (MPC) concept is an alternative in vitro measurement of drug susceptibility against the infecting pathogen, representing the drug concentration that prevents a population of more than 10^10^ colony forming unit (CFU)/mL bacteria from first mutation. Mutant selection window (MSW) is defined as the concentration range between MIC and MPC. The use of MPC and MSW is of aid in evaluating the capacity and potency of antimicrobial agents for the selection of resistant mutants [16].

Application of the MPC theory has been conducted in a variety of organisms associated with human and animals such as *Escherichia coli* [17], *Salmonella enterica* [18], *Staphylococcus aureus* [19]*, Pseudomonas aeruginosa* [20] and *Mannheimia haemolytica* [21]. Essentially, the concept of MPC has been historically and primarily established for fluoroquinolones because of the resistance mechanisms for antimicrobial agents and is applicable to other classes under some restriction at present [22]. So far, no published data of MPC have been available for *R.anatipestifer* yet. Therefore, the purpose of this study was to investigate the MPC of four antimicrobials against 105 *R. anatipestifer* isolates from China, providing advice on rational use of these antimicrobial agents, and further compare the potencies of these antimicrobials in selecting resistant *R. anatipestifer* mutants.

## Methods

### Bacterial strains

During the period of 2008 to 2014, we randomly collected *R. anatipestifer* isolates from the sick ducks or geese that exhibited typical symptoms of *Riemerella anatipestifersis* at the animal diagnostic departments of Guangdong Province, China. The clinical cases were provided by the farm owners who volunteered to participate in the study. A total of 105 *R. anatipestifer* clinical strains from ducks (*n* = 98) and geese (*n* = 7) were obtained and used in this study. The bacteria were identified by colony morphology and PCR method for partial sequence of outer membrane protein A as described previously [23]. The Animal Experimentation Ethics Committee of South China Agricultural University approved all experimental procedures. In principal, MPC measurement should be performed against organisms sensitive to antimicrobial agents (by MIC testing). Because no critical susceptibility breakpoints were available for these four compounds against *R. anatipestifer*, the resistance breakpoints were tentatively interpreted according to the Clinical and Laboratory Standards Institute (CLSI) recommendation for *E.coli* or *Pasteurella multocida* [24].

### Antimicrobial compounds

Antimicrobial agents exclusively approved for use in animals including ceftiofur, cefquinome, florfenicol, and tilmicosin were investigated in the present study. These compounds were commercially purchased from the manufactures in China. Stock solution of each antimicrobial was prepared in proper solvent according to the instructions of antimicrobial susceptibility testing for bacteria isolated from animals and stored at −20 °C. *Escherichia coli* ATCC 25922 and *Staphylococcus aureus* ATCC 29213 were used as control strains.

### MIC measurements

Minimum inhibitory concentrations were determined by CLSI agar dilution methodology [24]. All studies were carried out in triplicate. Briefly, each *R. anatipestifer* isolate in logarithmic growth period was diluted with 0.9 % saline to achieve a 0.5 McFarland standard suspension, equal to the inoculum of 5 × 10^5^ CFU/mL. About 5 μL suspensions were inoculated on Mueller-Hinton agar plates supplied with 5 % calf serum and containing antimicrobials (with a series of concentrations between 64 and 0.004 μg/mL). Inoculated plates were then incubated for 18 h at 37 °C in a constant temperature incubator. The MIC was recognized as the lowest antibiotic concentration showing no growth of colony morphology.

### MPC testing

The measurement of MPC was performed according to a method previously described with slight modification [21]. Briefly, three or four colonies were inoculated into 3 mL *R. anatipestifer* broth (tryptic soy broth containing 0.5 % yeast and 5 % new calf serum) and cultured overnight. The next day, 100 μL *R. anatipestifer* suspensions were transferred to 100 mL of *R. anatipestifer* broth and shaken at the speed of 200 rpm under the temperature of 37 °C overnight. The collected cultures were concentrated by centrifugation at 4000 rpm for 20 min at 4 °C and then re-suspended in 5 mL of fresh *R. anatipestifer* broth to produce ≥10^10^ CFU/mL suspensions. Aliquots of 100 μL containing ≥10^10^ CFU/mL were applied to *R. anatipestifer* agar plates incorporating a series of antimicrobials with the concentrations ranging from 512 to 1 × MIC. Each plate was prepared freshly, stored at 4 °C and used within 7 days. Inoculated plates were then incubated as described previously and observed for five days. MPC was taken as the lowest antimicrobial concentration that allowed no *R. anatipestifer* isolate growth. All MPC determinations were carried out in triplicate for each isolate. Results were identical and then used for data analysis. The ratio of MPC to MIC was also calculated.

## Results

### MIC and MPC

A total of 98 *R. anatipestifer* isolates from ducks and 7 field strains from geese were tested against ceftiofur, cefquinome, florfenicol and tilmicosin. MICs and MPCs of antimicrobials assayed are shown in Table 1. MIC_50/90_ and MPC_50/90_ values are also shown. MPC values were higher than MICs because of exposure to higher density of bacterial inoculum. Following testing, MIC values of ceftiofur ranged from ≤0.008 to 8 μg/mL, with MIC_50_ and MIC_90_ values of 0.063 μg/mL and 0.5 μg/mL respectively; cefquinome had the MIC values ranging between ≤0.008 to 16 μg/mL, with MIC_50_ of 0.031 μg/mL and MIC_90_ of 0.5 μg/mL respectively; florfenicol had the MIC values ranging from 0.125 to 16 μg/mL, with MIC_50_ of 1 μg/mL and MIC_90_ of 4 μg/mL respectively; tilmicosin had the MIC values ranging from 0.031 to 64 μg/mL, with MIC_50_ of 1 μg/mL and MIC_90_ of 4 μg/mL respectively.

**Table 1:** MIC/MPC distribution for four compounds with clinical isolates of *R. anatipestifer* (*n* = 105)

Drug	Distribution of MIC and MPC values (μg/mL)															
Concentrations	≤0.008	0.016	0.031	0.063	0.125	0.25	0.5	1	2	4	8	16	32	64	≥128	MIC_50_/MIC_90_
MIC distribution																
Ceftiofur	3	8	25	22	16	13	10	3	1	2	2					0.063/0.5
Cefquinome	28	22	15	6	12	9	5	3	3		1	1				0.031/0.5
Florfenicol					1	1	28	25	37	6	6	1				1/4
Tilmicosin			1	14	7	9	7	20	24	13	5	2	2	1		1/4
MPC distribution																MPC_50_/MPC_90_
Ceftiofur^a^					1	2	4	16	19	11	17	2	17	12	2	4/64
Cefquinome^b^						2	1	4	14	17	24	14	10	9	8	8/64
Florfenicol^c^								11	21	21	22	9	10	3	1	4/32
Tilmicosin^d^						2	1	7	4	10	22	10	10	5	31	16/256

MIC_50_ and MIC_90_ -the drug concentration at which 50 % or 90 % of the isolates are inhibited, respectively

MPC_50_ and MPC_90_-the drug concentration restricting the growth of mutant subpopulation for 50 % or 90 % respectively of the isolates tested

^a^ Testing against 103 isolates

^b^ Testing against 103 isolates

^c^ Testing against 98 isolates

^d^ Testing against 102 isolates

The corresponding MPC values of the four antimicrobial agents assayed against 105 *R. anatipestifer* isolates are also listed in Table 1. Following testing of ceftiofur, MPC values ranged from 0.125 to ≥128 μg/mL, with MPC_50_ and MPC_90_ values of 4 and 64 μg/mL respectively; for cefquinome from 0.25 to ≥128 μg/mL and 8 and 64 μg/mL respectively; for florfenicol from 1 to ≥128 μg/mL and 4 and 32 μg/mL respectively; for tilmicosin from 0.25 to ≥128 μg/mL and 16 and 256 μg/mL respectively.

Based on these MIC and MPC values, dosing to achieve the MIC or MPC values (where possible) may serve to inhibit susceptible bacterium or reduce the selection of resistant mutants. The hierarchy of potency for these tested agents based on MIC_90_ values was: ceftiofur = cefquinome > florfenicol = tilmicosin. The potency of antimicrobial agents tested based on MPC_90_ values followed the rank order: florfenicol > ceftiofur = cefquinome > tilmicosin.

### Mutant selection index calculations

The ratio of MPC to MIC was defined as selection index (SI) [25]. The lower SI is, the better ability of antimicrobials to restrict the resistant mutant subpopulations. Since working with a large population of *R. anatipestifer* isolates is cumbersome, we calculated the mutant selection index (ratio of MPC to MIC) for each isolate so that the capacity of selecting mutant enrichment for each antimicrobial agent could be easily compared. The value distribution for each antimicrobial agent was shown in Table 2. In our investigation, MPC/MIC ratios were slightly lower for florfenicol and higher for cefquinome. The SI data indicated a better ability of florfenicol to prevent non-susceptible mutant subpopulations and a strong selective pressure of cefquinome to enrich *R. anatipestifer* mutants.

**Table 2:** Distribution of different ratios of MPC to MIC for clinical isolates of *R. anatipestifer*

Drug	No. of isolates having different ratios of MPC to MIC										
	1	2	4	8	16	32	64	128	256	512	>512
Ceftiofur^a^	1	3	1	8	6	22	25	14	8	6	9
Cefquinome^b^	0	0	0	3	5	9	5	11	25	22	23
Florfenicol ^c^	6	25	30	18	12	5	2	0	0	0	0
Tilmicosin ^d^	1	5	15	20	8	17	14	10	5	3	4

^a^ Testing against 103 isolates

^b^ Testing against 103 isolates

^c^ Testing against 98 isolates

^d^ Testing against 102 isolates

### Pharmacokinetics/Pharmacodynamics (PK/PD) calculations

We also selected MIC_90_ and MPC_90_ as the boundaries of the mutant selective window [26]. Plasma pharmacokinetic data in ducks were available for cefquinome and florfenicol [27, 28]. In conjunction with pharmacokinetic parameters of each compound in ducks, the various PK/PD indices, including ratio of maximum plasma concentration to MIC_90_ (C_max_/MIC_90_) or MPC_90_ (C_max_/MPC_90_), ratio of area under the concentration-time curve to MIC_90_ (AUC/MIC_90_) or MPC_90_ (AUC/MPC_90_), time above MIC_90 _or MPC_90_ of the dosage interval ( %*T*>MIC_90 _or %*T*>MPC_90_), and time inside the mutant selection window of the dosage interval (%*T*_MSW_), are shown in Table 3. %*T* > MIC serves as an important parameter for cephalosporins and its value of cefquinome against *R. anatipestifer* isolates was approximately 31.67 % by integrating the pharmacokinetic values obtained from a intramuscular injection of a dose of 5 mg/kg body weight [27]. No concentration was observed to exceed the MPC_90_. According to the pharmacokinetic data attained with a single dose of 30 mg/kg body weight florfenicol intramuscularly [28], the values determined for C_max_/MIC_90_, C_max_/MPC_90_, AUC_24_/MIC_90_, AUC_24_/MPC_90_ were 1.62, 0.10, 18.21 h and 1.14 h for florfenicol, respectively. All the concentrations of florfenicol in plasma were lower than MPC_90_ and %*T*_MSW_ was calculated to be approximately 21.67 %.

**Table 3:** Pharmacokinetics and pharmacodynamics variables in plasma for two antimicrobial agents against *R. anatipestifer* isolates

Antimicrobial	Dosage regimen (mg/kg)	C_max_ (μg/mL)	AUC_24h_ (μg h/mL)	C_max_/MIC_90_	C_max_/MPC_90_	AUC_24_/MIC_90_ (h)	AUC_24_/MPC_90_ (h)	*T* > MIC_90_ (h)	*T* > MPC_90_ (h)	%*T*_MSW_
Cefquinome^a^	5, IM	9.38	23.78	18.76	0.15	47.56	0.37	~7.6	0	31.67
Florfenicol^b^	30, IM	3.24	36.42	1.62	0.10	18.21	1.14	~5.2	0	21.67

*C*_*max*_ serum maximum concentration, *AUC*_*24h*_ area under curve over a 24 h time period, *MIC* minimum inhibitory concentration, *MPC* mutant prevention concentration, *T*_*MSW*_ time inside the mutant selection window, *IM* intramuscularly

^a^ based on data as published by Yuan et al

^b^ based on data as published by EL-Banna

## Discussion

*Riemerella anatipestifer* has been a problematic pathogen of commercial importance for several years and it is hard to give the correct treatment measures. One possible reason is poor cross immune protection among various serotypes; another important reason may be due to the similarity of clinical symptom between *R. anatipestifer* infection and *E. coli* infection. In recent years, *R. anatipestifer* strains with reduced susceptibility to antimicrobials have emerged as reported in other’s study and our previous investigation [8, 23] because of the use of antimicrobials in animals, which reflects the necessity of searching for new compounds or strategies to treat *Riemerella anatipestifersis* [8, 10, 23]*.* Maintaining the utility of antimicrobials in treating *Riemerella anatipestifersis* seems to be a major challenge. As proposed by other researchers, one of the strategies to tackle the resistance problem is to reduce or prevent the emergence of resistant mutants [19]. In human medicine, MPC values and MPC/MIC ratios have been determined and studied extensively in a large number of antimicrobial agents, involving many kinds of organisms [29–32]. In the current study, we first report the MPC values of four antimicrobials that were exclusively developed for animals against 105 *R. anatipestifer* field strains mainly isolated from South of China.

We selected several antimicrobial agents that were approved exclusively and most commonly used in animals, and also represented a wide range of antimicrobial agents. To our surprise, up to 70 % strains had high MIC values of enrofloxacin (≥2 μg/mL) and apramycin (≥64 μg/mL) (data not shown). Based on the MIC data of the six compounds, four antimicrobial agents tested in our study appeared to have excellent in vitro activities against *R. anatipestifer* strains, although they have different chemical structures and action mechanisms. Previously, Blondeau et al. [21] compared the MIC and MPC values of five antimicrobial agents against bovine clinical isolates of *Mannheimia haemolytica*. The rank order of potency that antimicrobials selected resistant mutants differed by using MIC and MPC data. Such is the case for *R. anatipestifer* isolates.

The concentration zone between MIC and MPC was recognized as MSW. Concentrations of antimicrobials within MSW exerted a selective pressure for accumulation of resistant strains. Ratio of MPC to MIC represented the ability of antimicrobial agents to block the resistant mutant subpopulation. MPC, MSW and MPC/MIC ratio served as a guide for the potency of antimicrobials in restricting resistant mutant selection. Based on these, a large number of references tried to address the issue of relationship between antimicrobials and bacteria. Hansen et al. [20] thought ciprofloxacin was more active than levofloxacin in selecting resistant *Pseudomonas aeruginosa* amplification. By determining the MPC of three quinolones against 100 clinical Streptococcus *pneumococci*, moxifloxacin seemed to exhibit more excellent antimutant ability than levofloxacin and gemifloxin [33]. Wang et al. [34] tested the MPC of three quinolones against *Campylobacter jejuni* isolated from chicken and assumed that enrofloxacin had the lowest MPC among the three tested quinolones, thus enrofloxacin represented a low selective pressure for selection of resistant subpopulations. Briales et al. [35] provided the MPC values of fluoroquinolone against *E. coli* isolates carrying different plasmid-mediated resistant genes *qnr* and harboring isogenic *gyrA* and *parC* substitutions, considering that *qnr* genes played a vital role in selecting one-step resistant mutants. From our results, florfenicol appeared to be a compound with excellent in vitro activity against these *R. anatipestifer* strains collected from South China, although some mechanism of *R. anatipestifer* isolates resistant to florfenicol has been described in other districts [36].

The ratio of MPC/MIC was slightly higher for ceftiofur and cefquinome among the four antimicrobials tested (Table 2). Even two cephalosporins developed for animals are only used in veterinary medicine, and they are classified as critically important antimicrobials by the WHO [37, 38], their use to treat great numbers of animals in duck industry is to be strongly discouraged because of prudent use guidelines. Similar conclusion was also obtained for the therapy of *R. anatipestifer* infection based on the theory of MPC and MSW. In addition, both ceftiofur and cefquinome cannot be administered orally because of poor absorption; unless used prophylactically, which would be in strong contradiction to prudent use guidelines, therefore the usefulness of ceftiofur and cefquinome may probably be reduced in duck production to a bare minimum. Although ceftiofur has successfully cured the *R. anatipestifer* infection in a previous report [39], it should not be the first choice considering the wider MSW and severe drug resistance situation. The MSW of cefquinome was wider than that of ceftiofur. In other words, cefquinome has weaker ability of preventing the selection of *R. anatipestifer* mutants than ceftiofur. Comparing MSWs and MPC/MIC ratios of the four antimicrobials, cefquinome seems to be the drug that most easily selects resistant mutants.

MIC or MPC-based therapeutic protocols and PK/PD indices for suppressing the enrichment of resistant bacterial subpopulations have been proposed and studied in various in vivo or in vitro models extensively [40–43]. PK/PD parameters such as AUC/MIC, C_max_/MIC and T > MIC relate closely with the effect of antimicrobials. In our laboratory, pharmacodynamics on the basis of MIC values for cefquinome have been well studied in mice, yellow cattle, pigs and dogs against a series of microorganisms in recent years, involving *E. coli*, *S. aureus*, *P. multocida*, *Klebsiella pneumoniae* as well as *Haemophilus parasuis* [44–50]. These publications clearly described the relationship between the dosing schedules and the antimicrobial effectiveness. Also, the ability of cefquinome to restrict the selection of *E. coli* mutants was predicted in an in vivo model. The results demonstrated that %*T* > MPC of >50 % was favorable to block the resistant mutants [43]. By integrating our results with the published pharmacokinetic data of antimicrobials in ducks, serum drug concentrations of cefquinome and florfenicol may fall within the MSW and the high MPC values could hardly be attained albeit these two drugs had excellent MIC values. As cephalosporin exhibited time-dependent property, we applied this approach in our study and the predicted *T* > MIC was approximately 7.6 h (Table 3), which was lower than that obtained previously using *P. multocida* in yellow cattle [46], but slightly higher than that calculated using canine *E. coli* [50]. In vivo antimicrobial efficacy of cefquinome against *R. anatipestifer* should be further addressed. Little is known on the PK/PD relationship of florfenicol against *R. anatipestifer*. Until now, no killing studies of ceftiofur and tilmicosin based on MIC or MPC parameters of *R. anatipestifer* have been conducted in ducks. So more work based on the MSW theory should be performed for the use of antimicrobial agents in ducks.

## Conclusions

This is the first study that described the MIC and MPC values of four antimicrobial agents developed exclusively for animals against *R. anatipestifer* isolates. Our study may shed light on the future antimicrobial therapies for treatment of *R. anatipestifer* infection. Further in vivo or in vitro studies are required to confirm the efficacy based on the MIC or MPC values. The mutant selection window hypothesis suggests that cefquinome is least likely to prevent the emergence of *R. anatipestifer* mutants among the four antimicrobials.

## Abbreviations

CFU: Colony Forming Unit
CLSI: Clinical and Laboratory Standards Institute
MBC: Minimum Bactericidal Concentration
MIC: Minimum Inhibitory Concentration
MPC: Mutant Prevention Concentration
MSW: Mutant Selection Window
PD: Pharmacodynamics
PK: Pharmacokinetics
SI: Selection Index
